# Epitaxially Integrated Hierarchical ZnO/Au/SrTiO_3_ and ZnO/Ag/Al_2_O_3_ Heterostructures: Three-Dimensional Plasmo-Photonic Nanoarchitecturing

**DOI:** 10.3390/nano11123262

**Published:** 2021-11-30

**Authors:** Youngdong Yoo, Minjung Kim, Bongsoo Kim

**Affiliations:** 1 Department of Chemistry, Ajou University, Suwon 16499, Korea; 2 Department of Chemistry, KAIST, Daejeon 34141, Korea; mjmj0123@kaist.ac.kr

**Keywords:** epitaxial, hierarchical, heterostructure, three-dimensional, plasmo-photonic, nanoarchitecturing

## Abstract

In this study, we fabricated three-dimensional (3D) hierarchical plasmo-photonic nanoarchitectures by epitaxially integrating semiconducting zinc oxide (ZnO) nanowires with vertically oriented plasmonic gold (Au) and silver (Ag) nanoplatforms and investigated their growth mechanisms in detail. We synthesized 3D hierarchical Au–ZnO nanostructures via a vapor–solid mechanism leading to the epitaxial growth of ZnO nanowires on vertically oriented single-crystalline Au nanowires on a strontium titanate (SrTiO_3_) substrate. The elongated half-octahedral Au nanowires with a rhombus cross-section were transformed into thermodynamically stable elongated cuboctahedral Au nanowires with a hexagonal cross-section during the reaction. After the transformation, ZnO thin films with six twinned domains were formed on the side planes of the elongated cuboctahedral Au nanowire trunks, and six ZnO nanowire branches were grown on the ZnO thin films. Further, 3D hierarchical Ag–ZnO nanostructures were obtained via the same vapor–solid mechanism leading to the epitaxial growth of ZnO nanowires on vertically oriented Ag nanoplates on an aluminum oxide (Al_2_O_3_) substrate. Therefore, the growth mechanism developed herein can be generally employed to fabricate 3D hierarchical plasmo-photonic nanoarchitectures.

## 1. Introduction

Three-dimensional (3D) hierarchical nanoarchitectures have various exceptional physical and chemical properties owing to their unique structures; thus, they are used as advanced materials for various applications, including catalysis [1,2,3,4], optoelectronics [5], spintronics [6], and thermoelectrics [7]. In particular, 3D hierarchical nanoarchitectures in which all nanostructures are integrated epitaxially can exhibit innovative functionalities because dissimilar properties of different nanomaterials can be effectively combined due to perfect epitaxial bonding between nanomaterials. For instance, epitaxially integrated 3D hierarchical metal–semiconductor hetero-nanostructures can diversify the functionality of optoelectronic devices owing to the coupling of photons and plasmon [8,9], and they can be used as efficient materials for next-generation solar energy harvesting [10,11], photocatalytic hydrogen production [12], etc.

To date, various 3D hierarchical semiconductor nanostructures have been synthesized using one-step growth methods, such as screw-dislocation-driven growth, as well as two-step vapor–liquid–solid (VLS) growth methods in which catalytic nanoparticles are reseeded on the surface of as-synthesized nanostructures [13,14,15,16,17]. However, synthesizing epitaxially integrated 3D hierarchical metal–semiconductor hetero-nanostructures is quite challenging due to the lack of appropriate growth mechanisms. To the best of our knowledge, there is no report on the synthesis of epitaxially integrated 3D hierarchical metal–semiconductor hetero-nanostructures.

Metal–semiconductor hetero-nanostructures composed of gold/zinc oxide (Au–ZnO) and silver/zinc oxide (Ag–ZnO) exhibit much higher performance in photocatalytic degradation of organic contaminants than ZnO nanostructures due to plasmonic effects of Au and Ag [18,19,20]. Owing to the plasmonic effects, Au–ZnO and Ag–ZnO hetero-nanostructures also can possess higher photoelectric conversion efficiency than ZnO nanostructures in photovoltaic applications [21,22,23]. In addition, Au–ZnO and Ag–ZnO hetero-nanostructures can be used as highly efficient photocatalysts for hydrogen production reactions [24,25,26]. Although hetero-nanostructures composed of Au–ZnO and Ag–ZnO have been successfully synthesized for various applications as described above, the epitaxially integrated hierarchical 3D nanoarchitectures composed of Au–ZnO and Au–ZnO have not been reported yet.

Herein, we synthesized epitaxially integrated hierarchical 3D Au–ZnO and Ag–ZnO metal–semiconductor nanoarchitectures and investigated their detailed growth mechanisms. 3D hierarchical Au–ZnO nanostructures were synthesized by epitaxially growing ZnO nanowires on vertically oriented single-crystal Au nanowires via a vapor–solid (VS) mechanism. The geometry of the Au nanowires was changed from elongated half-octahedron with a rhombus cross-section to elongated cuboctahedron with a hexagonal cross-section during the reaction process. After twinned ZnO thin films were formed on the surface of Au nanowires, six ZnO nanowire branches were grown on the ZnO films. In addition, we synthesized 3D hierarchical Ag–ZnO metal–semiconductor nanostructures by epitaxially growing ZnO nanowires on vertically aligned Ag nanoplates via the same VS mechanism.

## 2. Materials and Methods

### 2.1. Preparation of Epitaxially Grown Vertical Au and Ag Plasmonic Nanoplatforms

To prepare epitaxially grown vertical Au and Ag plasmonic nanoplatforms, we used a seed-initiated growth mechanism [27,28]. Vertical Au nanowires and Ag nanoplates were synthesized in a 1 inch quartz tube using a horizontal hot-wall single-zone furnace equipped with a vacuum pump, pressure gauges, and mass-flow controllers. For the synthesis of vertical Au nanowires, Au slugs were placed in alumina boats in the middle of the heating zone, whereas Ag slugs were put in alumina boats in the middle of the heating zone for the synthesis of Ag nanoplates. After evacuating the quartz tube, carrier argon (Ar) gas flowed at a rate of 100 sccm to maintain a chamber pressure of 5–15 Torr. To synthesize Au nanowires and Ag nanoplates, the Au and Ag slugs were heated to 820 and 1100 °C, respectively. At the reaction temperatures, the Au and Ag vapors were transported to the lower-temperature region by the carrier gas where Au nanowires and Ag nanoplates were grown on SrTiO_3_ and Al_2_O_3_ substrates, respectively. The substrate sizes were chosen of 5 mm × 5 mm.

### 2.2. Synthesis of 3D Hierarchical Au–ZnO and Ag–ZnO Plasmo-Photonic Nanoarchitectures

Epitaxial ZnO nanowires were synthesized on vertically oriented plasmonic Au and Ag nanoplatforms in a 1 inch quartz tube using a horizontal hot-wall two-zone furnace equipped with mass-flow controllers. The reaction occurred at atmospheric pressure. Zn powder was placed in an alumina boat in the middle of the upstream zone, and the vertically oriented plasmonic Au and Ag nanoplatforms were located in the middle of the downstream zone. Ar and O_2_ gases flowed at a rate of 180 and 3 sccm, respectively. The upstream and downstream zones were heated to 750 and 450 °C, respectively. During the reaction, Zn vapor and O_2_ gas were transported to the vertically oriented plasmonic Au and Ag nanoplatforms where 3D hierarchical Au–ZnO and Ag–ZnO plasmo-photonic nanoarchitectures were synthesized, respectively.

### 2.3. Characterization

Field-emission scanning electron microscopy (SEM) images were taken using a Philips XL30S microscope (Stamford, CT, USA). The samples were coated with gold to reduce charging effects during SEM observation. Transmission electron microscopy (TEM), high-resolution TEM (HR-TEM) images, and selected-area electron diffraction (SAED) patterns were obtained using a TECNAI F30 TEM (Hillsboro, OR, USA) operated at 200 kV. TEM specimens were prepared by dropping a solution of nanostructures dispersed in ethanol on a holey carbon-coated copper grid. Cross-sectional TEM specimens were prepared by a dual-beam focused ion beam (FIB) equipped with a nanomanipulator.

## 3. Results

### 3.1. Vertical Au Nanowires and Three-Dimensional Hierarchical Au–ZnO Nanostructures

**Figure 1:**
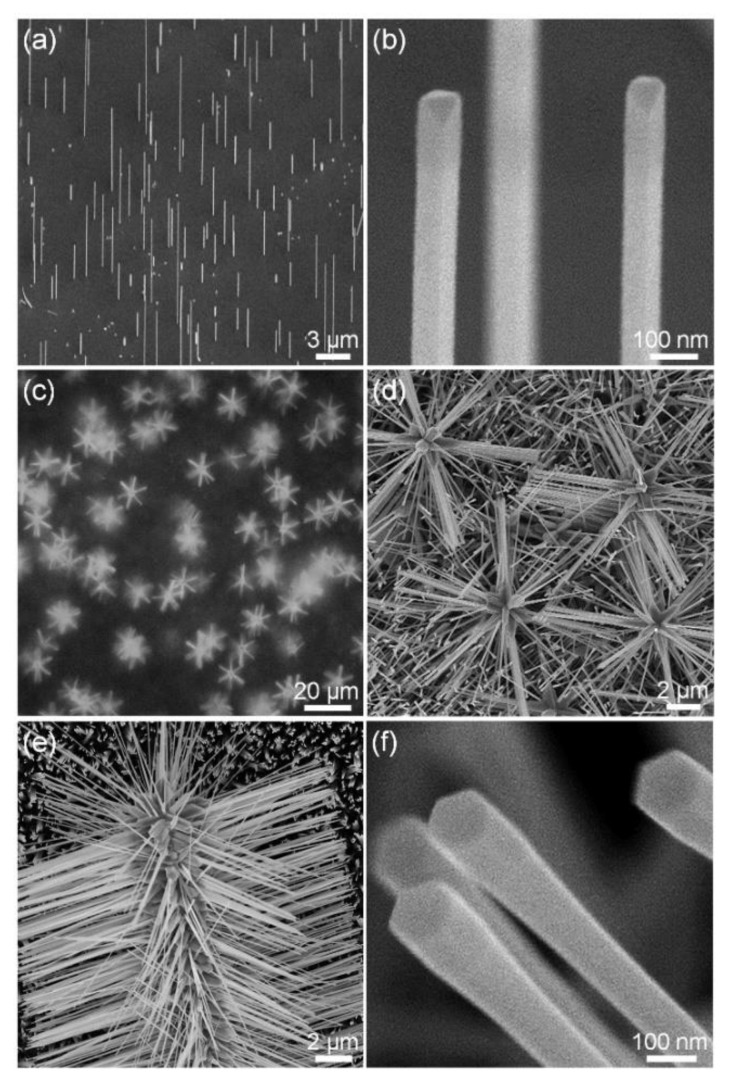
Vertically grown Au nanowires and epitaxially integrated 3D hierarchical Au–ZnO nanostructures. (**a**) 45°-tilted SEM image of Au nanowires grown vertically on a SrTiO_3_ (110) substrate. (**b**) 45°-tilted and magnified SEM image of the Au nanowires. (**c**) Top-view optical microscope image of 3D hierarchical Au–ZnO nanostructures. (**d**) Top-view and magnified SEM image of 3D hierarchical Au–ZnO nanostructures. (**e**) 45°-tilted and magnified SEM image of the 3D hierarchical Au–ZnO nanostructure. (**f**) 45°-tilted and magnified SEM image of ZnO nanowire branches.

To prepare vertically oriented plasmonic Au nanowire platforms, we directly synthesized Au nanowires on a SrTiO_3_ (110) substrate via a simple vapor transport method using Au slug as a precursor. As described in our previous paper, Au nanowires were grown vertically from half-octahedral Au seeds via the supply of Au atoms by direct impingement from a vapor at a low Au-atom flux condition [27]. A 45°-tilted SEM image (Figure 1a) shows that Au nanowires grew vertically in the same orientation. A 45°-tilted and magnified SEM image (Figure 1b) shows that the Au nanowires have an elongated half-octahedral shape. The elongated half-octahedral Au nanowires with rhombus cross-sections are single crystalline without twins, grew in the <110> direction, and are enclosed by {111} facets [27].

Using the as-synthesized vertical Au nanowires as a new substrate, we synthesized 3D hierarchical Au–ZnO nanostructures using a vapor transport method, in which Zn powder was used as a precursor, and O_2_/Ar gases were used as carrier gases. Figure 1c,d show the top-view optical and SEM images of vertically grown 3D hierarchical Au–ZnO nanostructures. ZnO nanowires grew in six directions on the side planes of the Au nanowires to form 3D hierarchical Au–ZnO nanostructures with a vertical orientation. Figure 1e shows a 45°-tilted and magnified SEM image of a 3D hierarchical Au–ZnO nanostructure. The ZnO nanowires have a hexagonal cross-section, and there were no catalyst particles at their tips (Figure 1f). In addition, TEM images further confirmed that there were no catalyst particles at the tip of the ZnO nanowires (Figure S1). Since no catalysts exist at the tip of the ZnO nanowires, we deduce that the ZnO nanowires were grown via a VS mechanism rather than a VLS mechanism [29,30,31].

### 3.2. Growth Process of Three-Dimensional Hierarchical Au–ZnO Nanostructures

**Figure 2:**
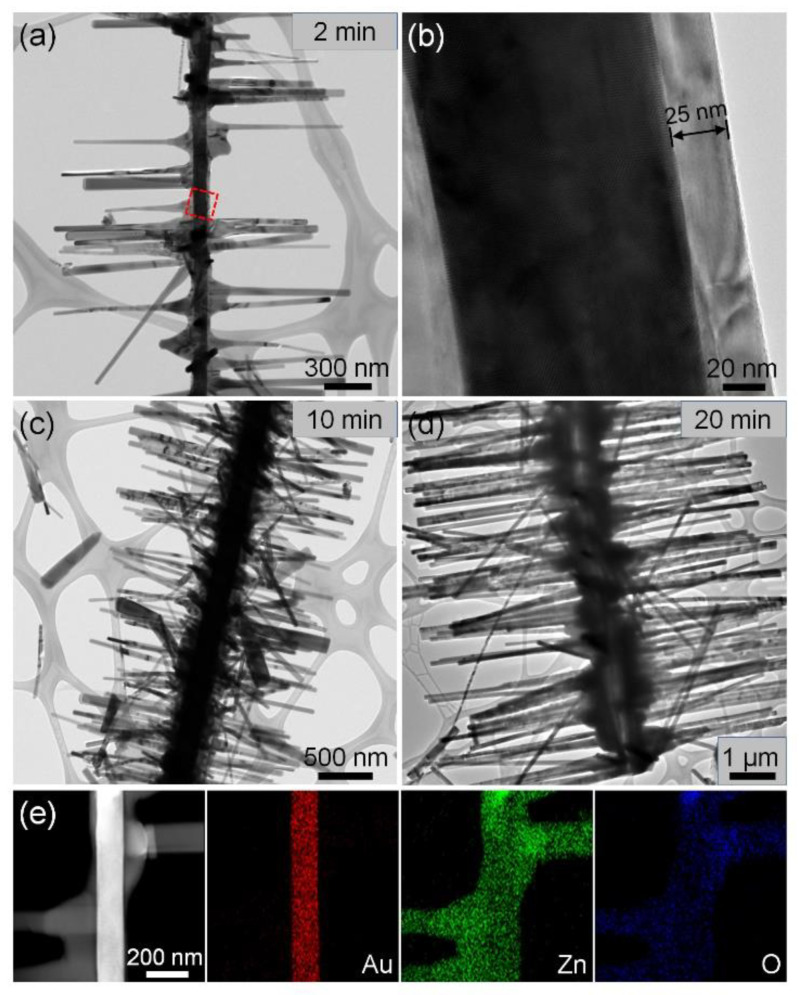
TEM analysis of 3D hierarchical Au–ZnO nanostructures. (**a**) TEM and (**b**) HR-TEM images of 3D hierarchical Au–ZnO nanostructure synthesized at a reaction time of 2 min. TEM image of 3D hierarchical Au–ZnO nanostructure synthesized at a reaction time of (**c**) 10 min and (**d**) 20 min. The ZnO films formed on the Au nanowires at reaction times of 2, 10, and 20 min were ~25, 100, and 300 nm thick, respectively. (**e**) TEM EDS elemental mapping images of 3D hierarchical Au–ZnO nanostructures.

To investigate the growth process of the 3D hierarchical Au–ZnO nanostructures, experiments were conducted for reaction times of 2, 10, and 20 min. The reaction time refers to the duration of maintaining the heating-zone temperature at the target temperature for the synthesis of ZnO nanowires. As-synthesized 3D hierarchical Au–ZnO nanostructures were characterized by TEM. When 3D hierarchical Au–ZnO nanostructures were synthesized at a reaction time of 2 min, ~25-nm-thick ZnO films were formed on the side planes of the Au nanowires, and ZnO nanowires with a length of ~800 nm were grown on the ZnO films (Figure 2a,b). When the 3D hierarchical Au–ZnO nanostructures were synthesized at a reaction time of 10 and 20 min, ZnO films formed on the side planes of the Au nanowires were ~100 and ~300 nm thick and ZnO nanowires grown on the ZnO films were ~1 and ~4 μm long, respectively (Figure 2c,d). The chemical composition of the as-synthesized 3D hierarchical Au–ZnO nanostructures was characterized by TEM EDS elemental mapping (Figure 2e), which showed that ZnO nanowire branches were formed on the thin ZnO films on the side planes of the Au nanowire trunk.

### 3.3. Internal Crystal Structure of Three-Dimensional Hierarchical Au–ZnO Nanostructures

**Figure 3:**
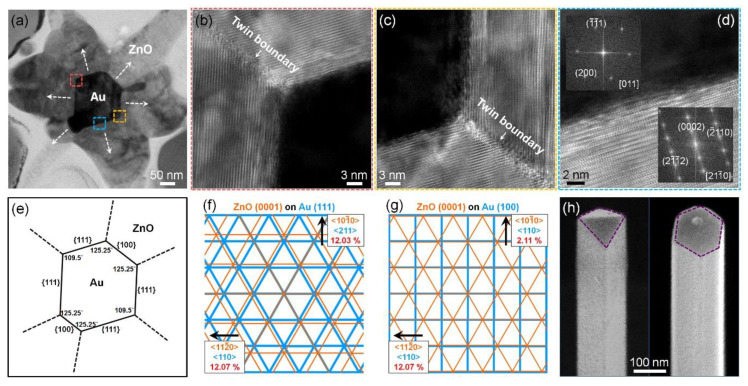
Cross-sectional TEM data of 3D hierarchical Au–ZnO nanostructures. (**a**) Cross-sectional TEM image of 3D hierarchical Au–ZnO nanostructures. (**b**) HR-TEM image of the red square in (**a**). (**c**) HR-TEM image of the orange square in (**a**). (**d**) HR-TEM image and FFT patterns (insets) of the sky-blue square in (**a**). (**e**) Illustration showing the internal crystal structure of the 3D hierarchical Au–ZnO nanostructures. (**f**) Schematic of atomic planes at the epitaxial interface between Au (111) and ZnO (0001). (**g**) Schematic of atomic planes at the epitaxial interface between Au (100) and ZnO (0001). (**h**) SEM image showing that the elongated half-octahedral Au nanowires were transformed into elongated cuboctahedral Au nanowires by thermal annealing.

To closely analyze the internal crystal structure of the 3D hierarchical Au–ZnO nanostructures, cross-sectional TEM measurements were conducted, which revealed that ZnO nanowire branches grew in six directions on the Au nanowires with a hexagonal cross-section (Figure 3a). Figure 3b,c shows magnified TEM images of the red dotted rectangle and orange dotted rectangle in Figure 3a, respectively. They show that ZnO films with twin boundaries were epitaxially grown on the side planes of the Au nanowires. Figure 3d shows an HR-TEM image and fast Fourier transform (FFT) patterns of the blue dotted rectangle in Figure 3a. The cross-sectional TEM analysis showed the internal crystal structure of the Au–ZnO nanostructures (Figure 3e). The Au nanowires showed an elongated cuboctahedral structure with six side facets consisting of {111} and {100} planes. On the side facets of the Au nanowires, thin ZnO films with six twinned domains were formed, and subsequently, ZnO nanowires grew in six directions from each ZnO domain. There are two epitaxial relationships between Au and ZnO, which are (111) Au//(0001) ZnO and Au (100)//(0001) ZnO. Since both Au (111) and ZnO (0001) planes have the same hexagonal symmetry, the ZnO (0001) plane can sit on the (111) plane of Au, despite the relatively large lattice mismatch of ~12% (Figure 3f). Although the symmetry of the ZnO (0001) plane differs from that of the Au (100) plane, the ZnO (0001) plane can sit on the Au (100) plane because the lattice mismatch between the ZnO (0001) and Au (100) planes is only 2.11% in the Au (110) direction (Figure 3g).

### 3.4. Proposed Growth Mechanism of Three-Dimensional Hierarchical Au–ZnO Nanostructures

Further, we investigated how the elongated half-octahedral Au nanowire with a rhombus cross-section changed into an elongated cuboctahedral Au nanowire with a hexagonal cross-section during the growth of ZnO nanowires. Since Au has a face-centered cubic structure, as-synthesized Au nanowires have an elongated half-octahedral shape enclosed by close-packed {111} planes, which are kinetically the most favorable. At a reaction temperature of ZnO nanowires (500 °C), the shape of Au nanowires changes from elongated half-octahedron to elongated cuboctahedron enclosed by {111} and {100} planes, which are thermodynamically the most favorable. From the atomic perspective, above a certain temperature at which atoms can migrate, atoms with the fewest nearest neighbor atoms migrate first because they are energetically the most unfavorable. Thus, atoms at the corners, where two {111} side planes meet in the elongated half-octahedral nanowires, in turn, migrate to form new {100} planes, resulting in a shape change from elongated half-octahedron to elongated cuboctahedron. Elongated half-octahedral Au nanowires were transformed into elongated cuboctahedral Au nanowires by annealing at 500 °C for 2 h (Figure 3h). Interestingly, during the synthesis of Au–ZnO nanostructures, the shape transformation of Au nanowires occurred for very short reaction times of 2–20 min. This is probably because the formation of ZnO overlayers promotes the transformation of Au nanowires. The formation of ZnO films with six twinned domains on elongated cuboctahedral Au nanowires would be more energetically advantageous than that on elongated half-octahedral Au nanowires with a rhombus cross-section.

Among the possible growth mechanisms of ZnO nanowires, we exclude screw dislocation-driven growth because no dislocation is observed in the ZnO nanowires (Figure S1). We also exclude vapor−liquid−solid (VLS) and vapor−solid−solid (VSS) growth because no catalyst is used. Thus, we deduce that the ZnO nanowires were grown via a vapor-solid (VS) mechanism. In order to observe very initial stage of the growth of ZnO nanowires, we synthesized 3D hierarchical Au–ZnO nanostructures at a reaction time of 0 min (Figures S2 and S3). Note that the reaction time of 0 min means that the reaction was terminated right after ramping to the target temperature. As-synthesized 3D hierarchical Au–ZnO nanostructures possess 3~5 ZnO nanowire branches (Figure S2). Interestingly, there still are thin ZnO films between Au nanowires and ZnO nanowires (Figure S3). Thus, we believe that the formation of ZnO films on Au nanowires is an important step for the growth of ZnO nanowires.

### 3.5. Vertical Ag Nanoplates and Three-Dimensional Hierarchical Ag-ZnO Nanostructures

**Figure 4:**
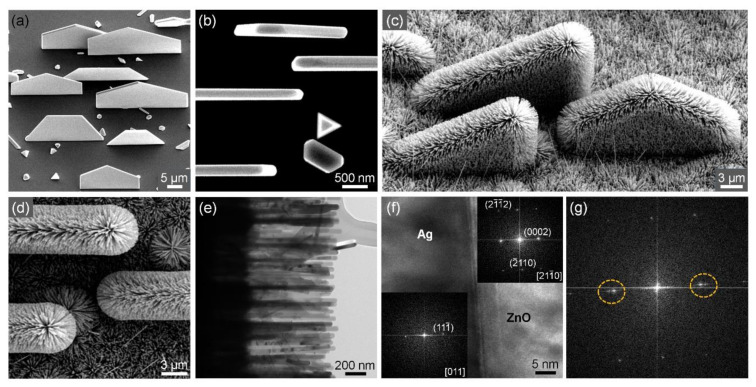
Vertical Ag nanoplates and 3D hierarchical Ag–ZnO nanostructures. (**a**) 45°-tilted SEM image of Ag nanoplates grown vertically on an r-cut sapphire substrate. (**b**) Top-view SEM image of Ag nanoplates. (**c**) 45°-tilted SEM image of 3D hierarchical Ag–ZnO nanostructures. (**d**) Top-view SEM image of 3D hierarchical Ag–ZnO nanostructures. (**e**) TEM image of 3D hierarchical Ag–ZnO nanostructures. (**f**) Cross-sectional TEM image and FFT patterns (insets) of 3D hierarchical Ag–ZnO nanostructures. (**g**) FFT pattern of the Au–ZnO interface in (**f**).

Such 3D plasmo-photonic nanoarchitecturing is generally applied to Ag nanoplates as well. As described in our previous paper, we synthesized Ag nanoplates on r-cut sapphire substrates via a simple vapor transport method using Ag slugs as a precursor (Figure 4a,b) [28]. The nanoplates were grown vertically in a single orientation in which the main facets are parallel to each other. The bottom side of the nanoplates was 20–25 μm thick and 120–250 nm long. When we synthesized ZnO nanowires using vertically grown Ag nanoplates as a new substrate, 3D hierarchical Ag–ZnO nanostructures were grown vertically in a single orientation on the substrate (Figure 4c,d). A TEM image of the Ag–ZnO nanostructure shows that the ZnO nanowire grew very densely and vertically on the Ag nanoplates (Figure 4e). The cross-sectional HR-TEM image and FFT patterns show that the epitaxial relationship between Ag and ZnO is (111) Ag//(0001) ZnO (Figure 4f). Figure 4g shows the FFT pattern at the Au–ZnO interface, and the yellow dashed circles indicate the (111) and (0002) diffractions of Ag and ZnO, respectively, confirming that ZnO nanowires were epitaxially grown on the Ag nanoplates.

### 3.6. Discussion on Potential Applications of Three-Dimensional Hierarchical Au–ZnO and Ag-ZnO Nanostructures

3D hierarchical Au–ZnO and Ag-ZnO nanostructures can be utilized for various applications due to their novel chemical and physical properties originating from their unique structure. One of the most promising applications of these 3D hierarchical Au–ZnO and Ag-ZnO nanostructures is the plasmon-enhanced photovoltaics and photocatalysis. We believe that 3D hierarchical Au–ZnO and Ag–ZnO nanostructures could possess much higher efficiency than ZnO nanostructures in photovoltaic and photocatalytic applications owing to the large roughness factor, fast electron transport, and the improved optical absorption arising from plasmonic effects of Au and Ag.

## 4. Conclusions

Herein, we synthesized 3D hierarchical Au–ZnO nanostructures by epitaxially integrating ZnO nanowires with vertical Au nanowires on a SrTiO_3_ substrate. During the reaction, kinetically the most favorable elongated half-octahedral Au nanowires with a rhombus cross-section were transformed into thermodynamically the most favorable elongated cuboctahedral Au nanowires with a hexagonal cross-section. After the transformation, ZnO thin films with six twinned ZnO domains were formed on the side planes of the elongated cuboctahedral Au nanowire trunks, and six ZnO nanowire branches were grown on the ZnO thin films. In addition, 3D hierarchical Ag–ZnO nanostructures were synthesized via the epitaxial growth of ZnO nanowires on vertically oriented Ag nanoplates on an Al_2_O_3_ substrate. This 3D plasmo-photonic nanoarchitecturing method can be employed generally to build 3D hierarchical plasmo-photonic nanostructures.

## Data Availability

Data presented in this article are available on request from the corresponding author.

## Supplementary Materials

The following are available online at https://www.mdpi.com/article/10.3390/nano11123262/s1, Figure S1: TEM images of 3D hierarchical Au–ZnO nanostructures; Figure S2: SEM images of 3D hierarchical Au–ZnO nanostructures synthesized at a reaction time of 0 min; Figure S3: TEM images of 3D hierarchical Au–ZnO nanostructures synthesized at a reaction time of 0 min.s.
