# Supplementary material for: Epitaxially Integrated Hierarchical ZnO/Au/SrTiO3 and ZnO/Ag/Al2O3 Heterostructures: Three-Dimensional Plasmo-Photonic Nanoarchitecturing
Source: Nanomaterials (Basel). 2021 Nov 30;11(12):3262. doi: 10.3390/nano11123262 (PMC8706281; doi:10.3390/nano11123262)
Supplement: Supplementary file 1 [file nanomaterials-11-03262-s001.zip › nanomaterials-1447273-supplementary.pdf]

*Supplementary Materials*

# Epitaxially Integrated Hierarchical $ZnO/Au/SrTiO_3$ and $ZnO/Ag/Al_2O_3$ Heterostructures: Three-Dimensional Plasmo-Photonic Nanoarchitecturing

**Youngdong Yoo [1,*], Minjung Kim [2] and Bongsoo Kim [2,*]**

[1] Department of Chemistry, Ajou University, Suwon 16499, Korea
[2] Department of Chemistry, KAIST, Daejeon 34141, Korea; mjmj0123@kaist.ac.kr
* Correspondence: yyoo@ajou.ac.kr (Y.Y.); bongsoo@kaist.ac.kr (B.K.)

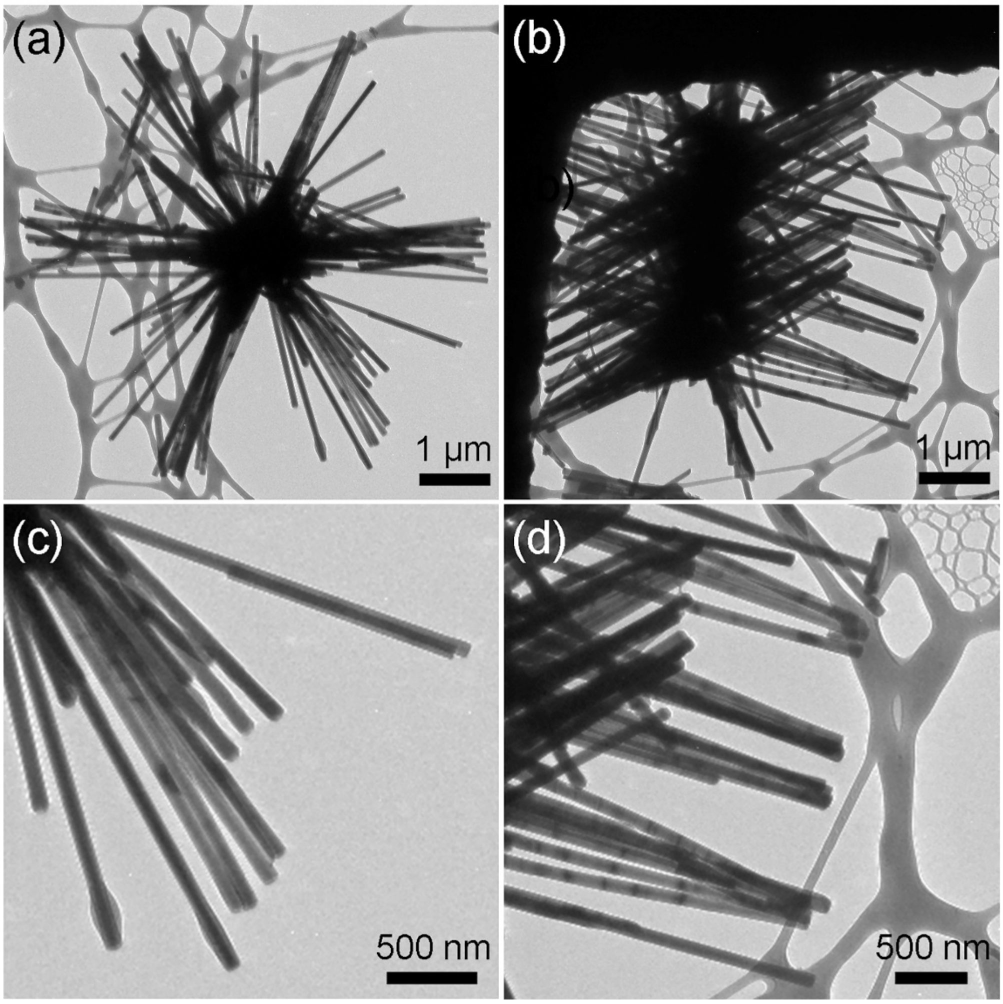


**Figure S1.** TEM images of 3D hierarchical Au–ZnO nanostructures. (a,b) Low-magnification TEM images of 3D hierarchical Au–ZnO nanostructures. (c,d) High-magnification TEM images of ZnO nanowire branches.

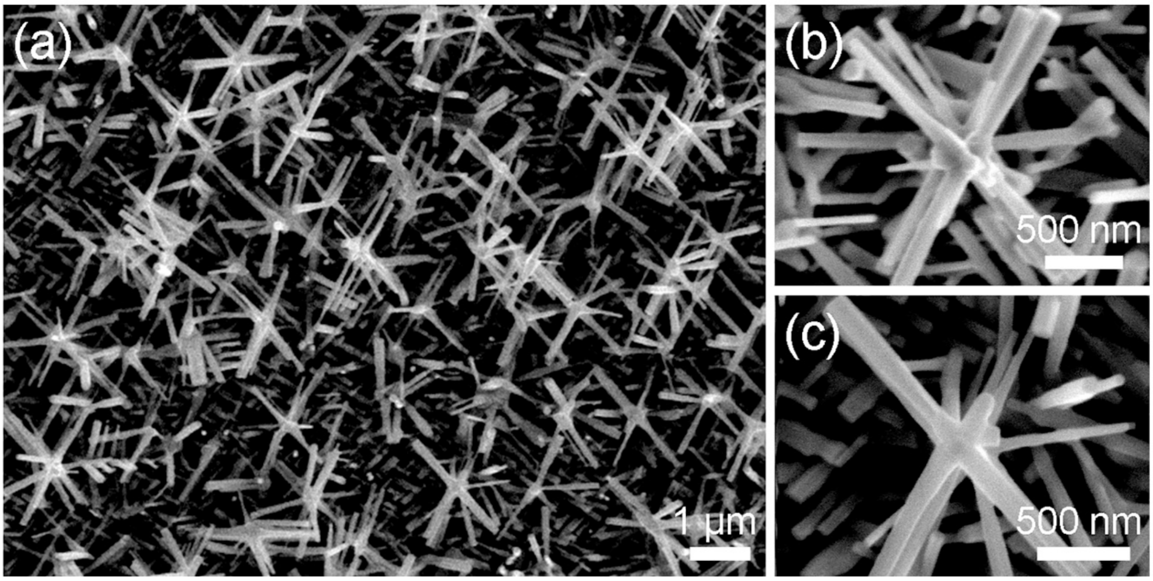


**Figure S2.** SEM images of 3D hierarchical Au–ZnO nanostructures synthesized at a reaction time of 0 min. (a) Low-magnification SEM images of the 3D hierarchical Au–ZnO nanostructures. (b,c) High-magnification SEM images of the 3D hierarchical Au–ZnO nanostructures.

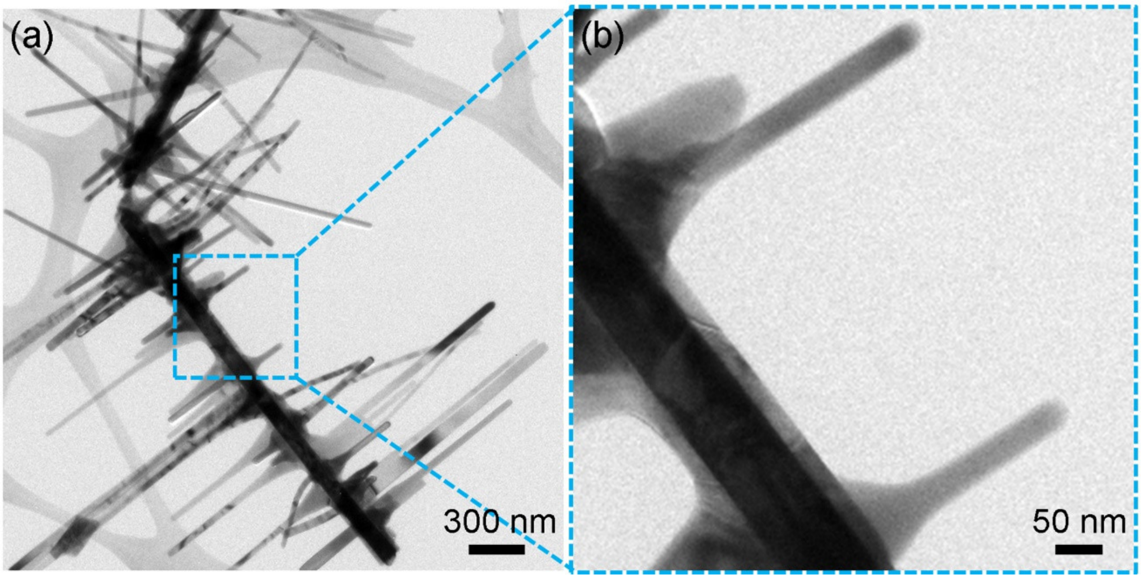


**Figure S3.** TEM images of 3D hierarchical Au–ZnO nanostructures synthesized at a reaction time of 0 min. (a) Low-magnification TEM image of the 3D hierarchical Au–ZnO nanostructures. (b) High-magnification TEM image of the 3D hierarchical Au–ZnO nanostructures.
